# Improving outcomes in adults with epilepsy and intellectual disability (EpAID) using a nurse-led intervention: study protocol for a cluster randomised controlled trial

**DOI:** 10.1186/s13063-016-1429-7

**Published:** 2016-06-24

**Authors:** Howard Ring, Nakita Gilbert, Roxanne Hook, Adam Platt, Christopher Smith, Fiona Irvine, Cam Donaldson, Elizabeth Jones, Joanna Kelly, Adrian Mander, Caroline Murphy, Mark Pennington, Angela Pullen, Marcus Redley, Simon Rowe, James Wason

**Affiliations:** Department of Psychiatry, University of Cambridge, Douglas House, 18d Trumpington Road, Cambridge, CB2 8AH UK; Cambridgeshire and Peterborough NHS Foundation Trust, Cambridge, UK; NIHR Collaboration for Leadership in Applied Health Research and Care (CLAHRC) East of England, Cambridge, UK; School of Health and Population Science, College of Medical and Dental Sciences, University of Birmingham, Edgbaston, Birmingham, B15 2TT UK; Yunus Centre for Social Business and Health, Glasgow Caledonian University, Cowcaddens Road, Glasgow, G4 0BA UK; King’s Clinical Trials Unit, PO64 Institute of Psychiatry, King’s College London, De Crespigny Park, London, SE5 8AF UK; MRC Biostatistics Unit Hub for Trials Methodology Research, Institute of Public Health, Robinson Way, Cambridge, CB2 0SR UK; Kings Health Economics, PO24, David Goldberg Centre, Institute of Psychiatry Psychology and Neuroscience, Kings College London, De Crespigny Park, London, SE5 8AF UK; Epilepsy Action, New Antsey House, Gate Way Drive, Yeadon, Leeds, LS19 7XY UK; NHS Leeds West Clinical Commissioning Group, Suites 2-4, Wira House, Wira Business Park, Leeds, LS16 6EB UK; NHS Wakefield Clinical Commissioning Group, White Rose House, West Parade, Wakefield, West Yorkshire WF1 1LT UK

**Keywords:** Epilepsy, Intellectual disability, Epilepsy nurse, Treatment, Quality of life, Cost-effectiveness, Clinical trial, Treatment as usual, Randomised, Clinical trials unit, Controlled trial, Nurse-led intervention, Cluster trial, Pragmatic

## Abstract

**Background:**

In adults with intellectual disability (ID) and epilepsy there are suggestions that improvements in management may follow introduction of epilepsy nurse-led care. However, this has not been tested in a definitive clinical trial and results cannot be generalised from general population studies as epilepsy tends to be more severe and to involve additional clinical comorbidities in adults with ID. This trial investigates whether nurses with expertise in epilepsy and ID, working proactively to a clinically defined role, can improve clinical and quality of life outcomes in the management of epilepsy within this population, compared to treatment as usual. The trial also aims to establish whether any perceived benefits represent good value for money.

**Methods/design:**

The EpAID clinical trial is a two-arm cluster randomised controlled trial of nurse-led epilepsy management versus treatment as usual. This trial aims to obtain follow-up data from 320 participants with ID and drug-resistant epilepsy. Participants are randomly assigned either to a ‘treatment as usual’ control or a ‘defined epilepsy nurse role’ active arm, according to the cluster site at which they are treated. The active intervention utilises the recently developed Learning Disability Epilepsy Specialist Nurse Competency Framework for adults with ID. Participants undergo 4 weeks of baseline data collection, followed by a minimum of 20 weeks intervention (novel treatment or treatment as usual), followed by 4 weeks of follow-up data collection. The primary outcome is seizure severity, including associated injuries and the level of distress manifest by the patient in the preceding 4 weeks. Secondary outcomes include cost-utility analysis, carer strain, seizure frequency and side effects. Descriptive measures include demographic and clinical descriptors of participants and clinical services in which they receive their epilepsy management. Qualitative study of clinical interactions and semi-structured interviews with clinicians and participants’ carers are also undertaken.

**Discussion:**

The EpAID clinical trial is the first cluster randomised controlled trial to test possible benefits of a nurse-led intervention in adults with epilepsy and ID. This research will have important implications for ID and epilepsy services. The challenges of undertaking such a trial in this population, and the approaches to meeting these are discussed.

**Trial registration:**

International Standard Randomised Controlled Trial Number: ISRCTN96895428 version 1.1. Registered on 26 March 2013.

**Electronic supplementary material:**

The online version of this article (doi:10.1186/s13063-016-1429-7) contains supplementary material, which is available to authorized users.

## Background

### Introduction – rationale for the trial

In adults with an intellectual disability (ID) and epilepsy there are suggestions that improvements may follow introduction of epilepsy nurse-led care. However, this has not been tested in a definitive clinical trial and results from previous studies within the general population cannot necessarily be generalised to adults with ID given the often relatively greater severity and complexity of epilepsy and associated morbidities in that group.

### Epilepsy and intellectual disability

Nearly one million adults in England have an ID and epilepsy is the most common medical illness in this group; affecting around 26 %, with higher rates in those with more severe ID [1, 2]. Individuals with ID have a worse outcome than those with epilepsy in the general population with increased seizure frequency, higher frequency of multiple anti-epileptic drug use and side effects, higher treatment costs, higher rates of mortality and greater incidence of behavioural problems [1, 3–5].

Reflecting these observations, it has been reported that between 2005 and 2009 the most common cause of avoidable acute hospital admissions for people with ID was seizures associated with poorly controlled epilepsy [6]. A survey by the Improving Health and Lives: Learning Disabilities Observatory (IHAL) reported that the second most frequent potentially preventable cause of death in those with ID was epilepsy or convulsions (13 %) [7]. These observations highlight the need to improve outcomes for people with epilepsy and ID.

Currently in the UK, secondary care of epilepsy and ID is generally provided by community learning disability services, or hospital-based neurological services [8]. It is common for people with ID to be on multiple therapies and they are likely to have tried several anti-epileptic medications to reduce seizure severity and frequency [3]. Only around 30 % of this population will achieve seizure freedom compared to 70 % of the general population [1, 4]. Therefore, in most people with epilepsy and ID, the aim of anti-epileptic medication is to reduce seizure severity and frequency whilst keeping associated side effects to a minimum [9]. Achieving this balance is often difficult in adults with ID due to the epilepsy’s severity and complex associated morbidities. Associated morbidities of epilepsy in adults with ID include difficulty understanding, mobility problems, communication difficulties, attentional deficits and a range of emotional, cognitive and behavioural problems [1]. Therefore identifying and differentiating presenting symptoms from the individuals’ ID, epilepsy and medication side effects can be problematic. There are often additional challenges for treating clinicians in communicating effectively with the individual with ID; putting them at ease and providing the most effective treatment and management [3], resulting in the highest possible overall quality of life for the individual. Unfortunately, epileptologists may be limited in being able to provide the necessary clinic time and often have limited training in ID [3]. Individuals with epilepsy and ID are also at greater risk of lacking capacity to understand and make decisions regarding their treatment, meaning that ‘best interest’ decisions may be required, often involving tripartite discussion between the individual, their family or paid carer and treating clinician [10]. This suggests that a multidisciplinary, holistic approach, with judicious use of expert nurses may improve support and access to healthcare in this group, who are often excluded from clinical research and overlooked by health services.

The aim of the trial described in this article is to test the hypothesis that an epilepsy nurse-led intervention is able to cost-effectively reduce seizure severity and improve overall quality of life for patients and those that provide care for them in the community.

### Epilepsy Specialist Nurses (EN) role

Epilepsy Nurses (ENs) offer a broad spectrum of services to patients with epilepsy. They contribute, depending on their level of training and expertise, to activities that may include patient assessment, medication management and ordering and interpreting investigations [11]. They also provide education, support and counselling to patients and families, often overlooked by other clinicians [12–14]. ENs may also have more time to speak to patients [15] and may improve continuity of, and accessibility to care, with the potential to improve communication between people with epilepsy and their primary healthcare services [16, 17]. Therefore, it is predicted that ENs would be ideally placed to champion the unpredictable, complex and long-term needs of people with epilepsy [18].

However, a Cochrane review [19] of five trials of epilepsy nurse specialists found no convincing evidence across the general population that ENs improve overall outcomes for people with epilepsy. Nevertheless, a recent open prospective survey, published after the Cochrane review, of the effects of introducing a paediatric EN, suggested ENs might reduce emergency admissions by as much as 50 % [20]. Thus a state of clinical equipoise may be considered to exist with respect to the question of whether it would be effective and cost-effective to systematically employ ENs to manage epilepsy in adults with ID.

In terms of financial costs, a trial reported by Meads et al. [21], in patients recruited from a hospital-based epilepsy service of whom just under 10 % had an ID, noted that the use of an epilepsy nurse cost less than standard care, with reduced numbers of outpatient clinic hospital attendances with doctors and a potential decrease in GP consultations after 6 months.

The great majority of previous research into the use of ENs has been in the general population, in studies that have completely or largely excluded adults with ID. However, it is known that people with ID have been subject to a range of health inequalities. Advocacy organisations and especially Mencap [22] have highlighted inequalities in healthcare noting that clinicians regularly fail people with ID - failing to consult with or involve parents and family members in care and treatment decisions. Despite these observations, a survey of epilepsy services for a community sample of adults with ID and epilepsy [23] found that just 34 % of respondents had seen an EN. This low exposure rate is despite the perception that ENs may enhance the care of people with ID and epilepsy [24–26].

### The Epilepsy and Intellectual Disability (EpAID) Trial - aim

The EpAID Trial aims to address two specific objectives. The primary objective is to establish whether nurses with expertise in epilepsy and ID, working to a clinically defined role outlined by The Learning Disability Epilepsy Nurse Competency Framework [27], can reduce seizure severity in adults with ID compared to treatment as usual.

The secondary objectives are to establish whether any observed benefits of clinical and quality of life outcomes justify any additional resource requirements, from the perspective of Health and Social Services.

## Methods/design

### Primary objective

To establish whether nurses with expertise in epilepsy and intellectual disabilities (ID), working to a defined clinical role, can improve epilepsy-related clinical and quality of life outcomes in the management of epilepsy in adults with ID compared to treatment as usual.

### Secondary objective

To establish whether any clinical benefits found justify the provision of additional resources associated with EN input after consideration of the costs associated with the intervention from the perspective of Health and Social Services. Additional secondary outcomes comprise measures of carer strain and quality of life to examine how the use of the competency framework, compared to treatment as usual, impacts on relationships critical in delivering ongoing care for adults with ID and epilepsy.

### Trial design

**Fig. 1:**
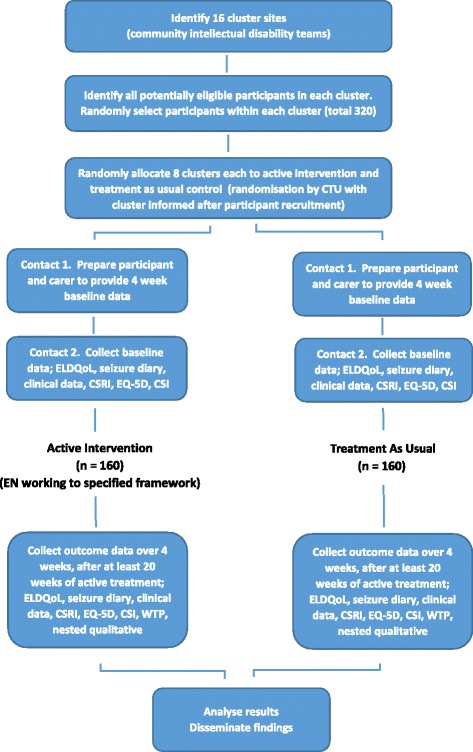
EpAID trial design. Trial design including the measures to be obtained. *CIDT* community intellectual disability team, *CSI* Carer Strain Index, *CSRI* Client Service Receipt Inventory, *CTU* Clinical Trials Unit, *ELDQoL* Epilepsy and Learning Disabilities Quality of Life scale

**Fig. 2:**
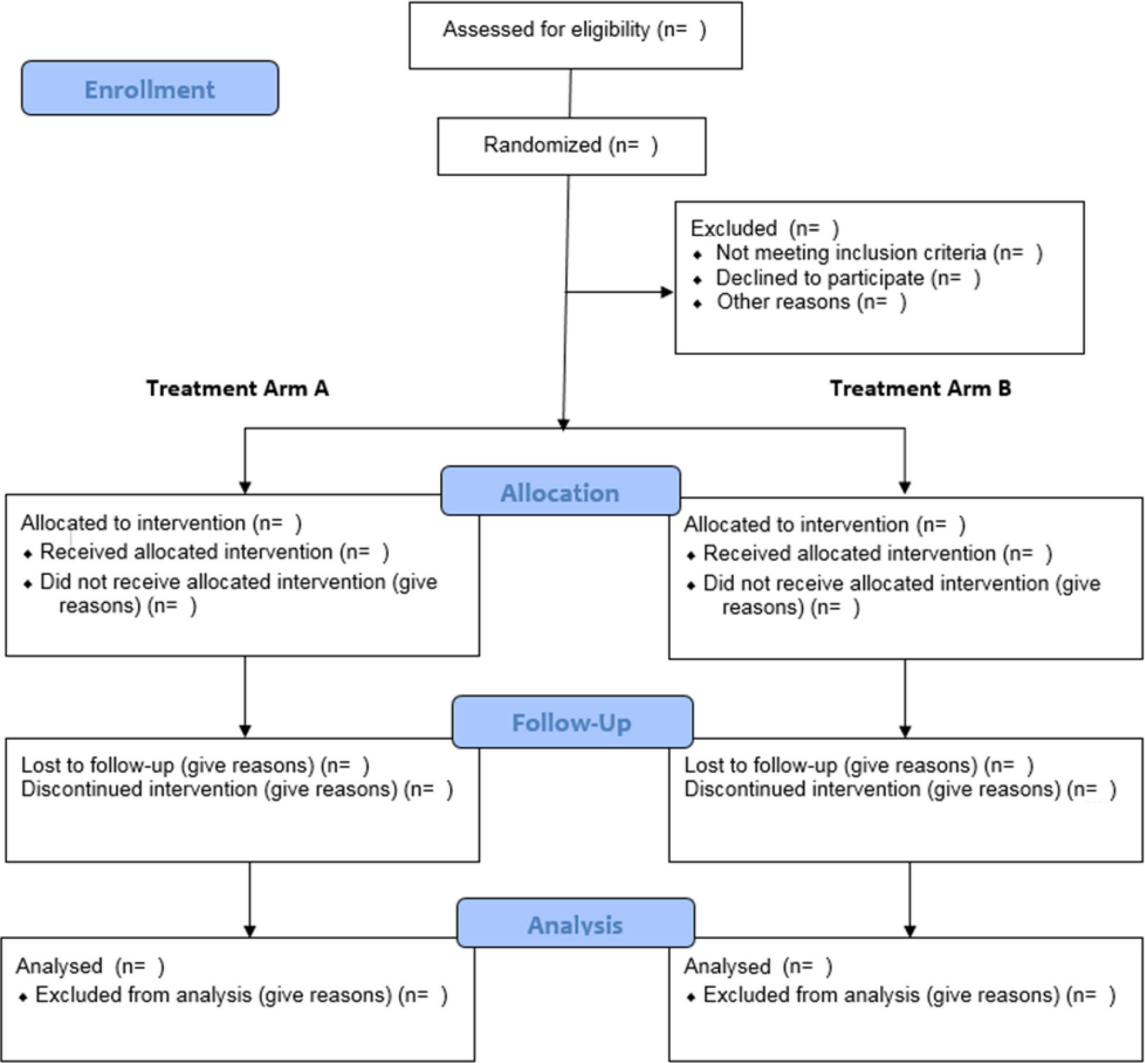
CONSORT diagram. A blank CONSORT diagram to record the number of participants enrolled, number allocated to treatment arm, number lost to follow-up and number analysed

**Fig. 3:**
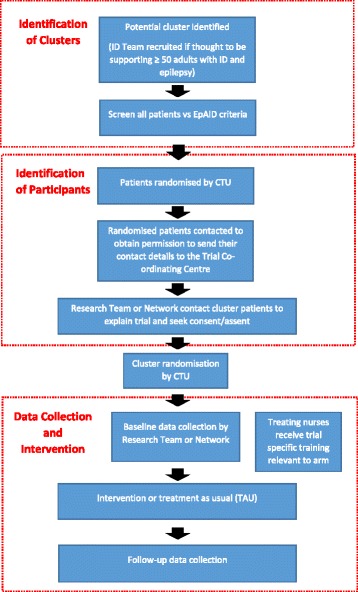
SPIRIT EpAID trial process flow diagram. Flow diagram describing the order in which cluster and participant recruitment, cluster randomisation, data collection and intervention processes at each research cluster took place

The study is a cluster randomised trial with two arms; a ‘treatment as usual’ control and a novel ‘defined epilepsy nurse (EN) role’ trial arm, complying with CONSORT guidelines for cluster randomised trials [28]. The study also contains a nested qualitative component. The design of the trial can be seen in Fig. 1 and the CONSORT diagram in Fig. 2. A SPIRIT flow diagram describing the order in which cluster and participant recruitment, cluster randomisation, data collection and intervention processes at each research cluster took place is provided in Fig. 3. A populated SPIRIT checklist is available as an Additional file 1.

Randomisation of the clusters is undertaken independently by a Clinical Trials Unit (CTU) supporting the trial, using block randomisation with fixed block sizes (in pairs).

The trial is pragmatic in design and formal blinding around this intervention is not possible. However, steps have been taken to minimise risks of bias developing, as described in the appropriate section below.

## Methods: participants, interventions, outcomes

### What does the trial involve?

The trial compares the effectiveness of the Epilepsy Nurse (EN) intervention to treatment as usual in 16 community intellectual disability (ID) services. The EN intervention comprises individually focused active support and management by an epilepsy nurse working according to a specific set of guidelines developed by the UK Epilepsy Specialist Nurse Association in association with the UK Royal College of Nursing [27]. The treatment as usual condition comprises the existing management approach, which is likely to include some combination of input by ID psychiatrists, neurologists, ID nurses and specialist epilepsy nurses.

Each participant undergoes 4 weeks of baseline data collection, followed by a minimum of 20 weeks intervention (novel treatment or treatment as usual), followed by a 4-week period of follow-up data collection. Once the follow-up data has been obtained for all participants in a cluster, the study will end for that cluster. Patient recruitment for the first trial site commenced in October 2014.

In addition, a carer and a nurse treating a nested subsample of up to 30 participants from each arm undergo a qualitative interview, held during the follow-up period. Six clusters from each arm are randomly selected by the CTU, in each of which up to five carers and five nurses are interviewed. These interviewees are not randomly selected but are a convenience sample.

### Eligibility criteria

Inclusion criteria:

1. Age 18–65 years.
2. The presence of a developmental intellectual disability with IQ of 70 or less.
3. A diagnosis of epilepsy with a history of at least one seizure in the 6 months preceding recruitment into the study (not considered by those managing the epilepsy to have been a non-epileptic seizure).
4. A nurse in the Community Intellectual Disability Team has a current role in delivering some aspects of epilepsy management at the time of both screening and consent.

Exclusion criteria:

1. The presence of a rapidly progressive physical or neurological illness.
2. Alcohol or drug dependence.

### Intervention

ENs in the intervention arm engage in the following activities [27]:

- Establish a relationship with patient and carers with regular collection of clinical information including seizure frequency, side effects, behavioural symptoms and effects of seizures on daily life from patient and carers. ENs also assess the patient on a regular basis at a frequency determined by clinical need. This is achieved through home visits, telephone clinics and visits to the local primary care or ID team base as appropriate. ENs also help manage the epilepsy of the participant and associated complications, as well as providing epilepsy education to patients and carers. ENs are the first point of contact for the service user/carer. The intervention takes place in the community, delivered face-to-face and by telephone with participants in their own homes and other community settings, such as day care facilities, as appropriate.
- Relationships with other clinicians are maintained by ENs facilitating well-informed assessments by participants’ primary care health service, local community ID health team and/or local neurology service as required.

The ‘treatment as usual’ comparator:

- Participants receive the same management as they did prior to entering the study. This varies throughout the country and includes various combinations of input by ID psychiatrists, neurologists, ID nurses and epilepsy nurses, often following locally developed care pathways. The nature of the ‘treatment as usual’ is described during the baseline phase of the trial by the use of a community ID team epilepsy service questionnaire (Community Intellectual Disability Epilepsy-Service Availability Questionnaire), and throughout the trial by use of the EN self-completion activity diary [29]. The ENs are responsible for completing the above questionnaire and the diary in both arms of the trial.

#### Training to be received by those delivering treatments

- All nurses receive training for diary completion by experienced educationalists/epilepsy specialist nurses. All ENs also have their clinical practice during the trial monitored by completing the aforementioned diary on a daily basis. The EN self-completion diary provides a reliable account of EN practice at a relatively low cost [30] and has been used successfully as a data collection method in a number of EN studies [31–33]. It is anticipated (from previous studies) that diary entry should take ENs approximately 15 minutes each day.

#### Contextual information

- The level of therapist competence at each research cluster is determined at baseline prior to the start of the trial according to the criteria described in the Learning Disability Epilepsy Nurse Competency Framework [27].
- The resources available locally at each cluster for providing epilepsy treatment to adults with an ID are determined at baseline prior to the start of the trial using a Community Intellectual Disability Epilepsy-Service Availability Questionnaire.
- The work undertaken by ENs during baseline is determined during the trial using an EN self-completion activity diary, which is completed throughout the period that the trial lasts at each cluster.

### Outcome measures

#### Primary outcome

- The primary outcome measure is the severity of seizures following intervention compared to treatment as usual. Seizure severity is measured using the Seizure Severity Scale from the Epilepsy and Learning Disabilities Quality of Life instrument (ELDQoL-SSS) [34]. The measure is completed at the end of the 4-week baseline period and again after 24 weeks in the trial. The baseline period refers to the 4 weeks prior to treatment beginning (whether this be continuation of treatment as usual or the beginning of the nurse-led intervention). The ELDQoL-SSS is designed for use as a proxy measure; it is completed by carers and provides a detailed measure of the physical severity of seizures experienced in the preceding 4 weeks, including any associated injuries and the level of distress manifest by the patient after a seizure.

#### Secondary outcomes

The secondary outcome measures include an economic evaluation of the EN intervention. This takes two forms: a cost-utility analysis based on utility data collected using the EQ-5D-5 L and a cost-benefit analysis in which benefit is assessed by contingent valuation of the service by carers. Differences in resource use are estimated using a modified Client Service Receipt Inventory [35], collected over the baseline period and again for 4 weeks after 24 weeks of intervention. The majority of the Client Service Receipt Inventory questionnaires are collected from paid carers and family carers, with occasional input from participants where a carer is not available. Time diaries are completed by ENs.

Additional secondary outcome measures:

- A contingent valuation of current practice is conducted. Maximum willingness-to-pay values for support from ENs (from carers in both arms) provides a measure of the strength of preference across the two service options, collected after at least 24 weeks in the trial. This instrument is only employed when the participant’s main carer is a family member, rather than a paid support worker.
- The effect of the intervention on carers and patients, measured using the Carer Strain Index [36], which is collected in the baseline period and after at least 24 weeks in the trial.
- The EQ-5D-5L [37], which permits estimation of changes in health-related quality of life attributable to the intervention and comparison with the primary outcome measure (ELDQoL-SSS) in terms of sensitivity to change.
- Seizure frequency is derived from entries by the participant’s carer in a seizure diary. Carers are asked to update this diary on a daily basis, reporting all seizures experienced by participants during the baseline and follow-up periods. Seizure frequency data records the number of types of seizures alongside the numbers of each type of seizure.
- Occurrence of possible anti-epileptic drug (AED) adverse effects is assessed using the ELDQoL questionnaire for the 4-week baseline period and again for 4 weeks after 24 weeks of intervention.
- Mood and behaviour outcomes are also assessed using the relevant ELDQoL subscales.
- A series of qualitative semi-structured interviews of samples of clinicians, family and paid carers are undertaken during the follow-up period. These examine how the competency framework, compared to treatment as usual, impacts on relationships between the EN and family/paid carers with respect to their reported perceptions of patient health and quality of life, the involvement of patients in treatment decisions and the active engagement of carers with clinical epilepsy services.

#### Descriptive measures

Demographic and clinical descriptors of participants are collected from participants’ clinical notes at baseline. The descriptors include the level of ID, nature of accommodation (also collected at follow-up), gender, age, current anti-epilepsy treatment (also collected at follow-up), and any additional ID syndrome, psychiatric and neurological diagnoses.

### Sample size for quantitative data collection

Data from an earlier study (LD-ROME) examining the use of the ELDQoL in adults with an ID [8] were used to estimate the interclass correlation coefficient (ICC) and individual level standard deviation for the seizure severity subscale of the ELDQoL outcome (ELDQoL-SSS). The estimated ICC was near to 0, but with a wide confidence interval. The estimated standard deviation of the change in ELDQoL-SSS outcome from that study was 6.55 [8]. It was chosen to power the study for the change between the baseline and follow-up of the ELDQoL-SSS outcome assuming an ICC of -0.05, which was above the estimated value in LD-ROME.

Assuming the difference in ELDQoL-SSS at baseline and after 24 weeks has a standard deviation of 6.55 in both treatment arms, and an ICC of 0.05, 16 clusters of 15 patients each would give 86 % power, at a one-sided 0.025 significance level, to detect a true mean intervention effect of 3.6. Recruiting 20 patients per cluster allows for the potential dropout rate of over 10 % (calculated based on the LD-ROME outcome), without impairing the statistical power of the data analysis.

### Sample size for the qualitative data collection

Sixty pairs of interviews, with carers and clinicians, are conducted. Participants are determined pragmatically on the basis of availability, with the aim of including patients living in a range of social circumstances.

### Recruitment

#### Participants and recruitment

We aim to recruit 20 participants from each of 16 different community ID services, each of which comprises a single research cluster, providing a total of 320 participants. The recruitment process is summarised in Fig. 3.

Initially, potential sites, identified by the trial research team or through local research networks, are contacted to enquire whether they are willing to participate in the trial if they meet the criteria of employing one or more nurses who delivers at least some aspects of epilepsy management to patients with ID and epilepsy and if they have at least 50 current patients with ID and epilepsy. Following recruitment of a site, members of the direct clinical care team supported, where appropriate permissions have been gained, by nurses from the local clinical research network, screen all potential participants. An anonymised list of eligible potential participants is sent to the clinical trials unit (CTU) supporting the trial, who provide the research team with a sequence in which to contact them. The first 34 potential participants are contacted in the first instance. If any potential recruit does not wish to participate then the next person in the sequence is approached. Participant recruitment is completed before site randomisation.

## Methods: assignment of interventions

### Group allocation

#### Sequence generation

Randomisation of the clusters is undertaken independently by the CTU using block randomisation with fixed block sizes. A minimum of two sites are randomised at a time to preserve allocation concealment. Site details are submitted to the CTU after potential participants within the centre have been identified and the randomisation outcome is confirmed to relevant members of the study team. Cluster randomisation to treatment arm takes place close to the start of the intervention phase to minimise the risk of clusters withdrawing between randomisation and the start of the trial.

#### Concealment mechanism

The nature of the active intervention, being a change in working protocols for the nurse, is such that the study cannot be fully blind. However, measures are taken to minimise the risk of bias being introduced. These are listed below:

- There is random selection of participants into the trial at each cluster and random allocation of each cluster to treatment arm.
- Individual cluster sites are not informed of the intervention they are to deliver until the month prior to the intervention phase and only after participant recruitment at that site is completed.
- In order to minimise expectations of the participants, their carers and families and the clinical staff at each cluster, they are not informed that there are two arms to the trial – ‘active’ and ‘treatment as usual’. Nor are they informed which arm of the study they are in. They are told instead about the range of interventions included in the trial and that their treatment may or may not change.
- It is expected that the ongoing management variability that is often a feature of management of drug-resistant epilepsy will blur perceived variations in management associated with the two arms of the study. Nevertheless, it is possible that some participants and their carers/paid support workers may notice differences in the management they receive during the trial.
- The people involved in the research informed of which arm a cluster has been randomised to are staff at the CTU undertaking the randomisation, the academic nurse providing training to the ENs delivering the trial interventions, and the Chair of the Trial Data Monitoring and Ethics Committee (DMEC) if requested. All other members of the research team remain blind to which arm a cluster has been randomised into.
- Data collection by researchers is via telephone or at participants’ homes/day placements. The nurses delivering the treatment interventions are not present at these assessments.
- The primary and the majority of the secondary outcome measures take the form of structured questionnaires and respondents are asked to consider their responses to these questions before reporting them to the researchers collecting the data.

## Methods: data collection, management, analysis

### Data collection methods

Obtaining patient consent, together with collection of patient baseline and follow-up data is undertaken by Research Assistants employed by the University of Cambridge and in some cases by Research Network Nurses at the trial cluster sites. The majority of the data collection is undertaken over the telephone by Research Assistants. Research Assistants mostly talk to carers of participants, due to the nature of the questions being asked. This also bypasses any issues which may arise for participants whose level of intellectual disability is too severe for them to provide details for the data collection. The use of telephone interviews allows a more convenient and low-cost method of data collection than face-to-face interviews. Participants/carers are sent the questionnaires in advance of the telephone call and then their answers are noted by the research assistant during the prearranged talk. Qualitative interviews are conducted and audio-recorded over the telephone for later transcription. A willingness-to-pay health economic questionnaire is also collected over the phone during the follow-up phase.

### Data management

The trial employs an electronic case report form (eCRF) created using the InferMed Macro database system. Data is managed via this system. The eCRF is created in collaboration with the trial statistician and the chief investigator and maintained by the CTU. It is hosted on a dedicated secure server within the CTU’s host academic institution. The system is regulatory compliant (good clinical practice (GCP), 21CRF11, EC Clinical Trial Directive) with a full audit trail, data discrepancy functionality, database lock functionality, and supports real-time data cleaning and reporting.

The trial manager is responsible for requesting usernames and passwords via the CTU for permitted local study personnel. There are different permission levels given to the system by virtue of individual usernames. Only those authorised by the trial manager are able to use the system. Paper CRFs are available as a back-up.

### Quality assurance

The study incorporates a range of data management quality assurance functions. The eCRF system contains a range of validations that alert sites to inconsistencies in the data being entered, which is monitored by the trial manager. The trial manager provides relevant staff training, ongoing study support and monitors study data as it accumulates, checking source data for transcription errors. Any necessary alterations to entered data are date and time stamped within the eCRF.

The monitoring and data management plan are updated as the trial progresses, detailing the quality control and quality assurance checks to be undertaken.

All members of the research team who have direct contact with participants have received good clinical practice training and informed consent training. Further, questionnaires used in the study are validated and references can be found in the ‘measures’ section.

## Statistical methods

### Data analysis

Data is entered electronically into an online database on a secure server. No patient is identifiable from the information recorded about them. Paper copies of data collection tools are kept in a locked filing system with restricted access at the University of Cambridge.

#### Clinical outcome analyses

The difference in treatment outcome between the competency framework arm and the treatment as usual arm is calculated as the difference between the baseline and follow-up ELDQoL-SSS measurement. A linear mixed-effects model is used with a random effect for cluster to account for the trial being cluster randomised. Baseline patient covariates to be included as fixed-effects are ELDQoL-SSS, age, level of intellectual disability (mild, moderate, severe or profound), mean number of seizures per month averaged over the 4 weeks preceding entry to the trial, and living circumstances (independent; with family; in group home). Cluster level covariates included as fixed-effects are the deprivation index of the cluster area, seniority level of the nurse (‘novice’, ‘competent’ or ‘expert’) to investigate possible therapist effects, and overall caseload of the nurse (number of patients). Whether a nurse is a nurse-prescriber is also recorded. The seniority criteria are such that a nurse-prescriber is rated as ‘expert’. However, preliminary data indicate that fewer than 10 % of nurses are prescribers and it is unlikely that prescriber status will be independently considered as a covariate. A Wald test for effect of intervention is used as the primary analysis.

Missing data on the ELDQoL-SSS are dealt with using a missing at random assumption. Where >3 % of data are missing, multiple imputation is used for the primary analysis. Five imputations are used unless the percentage missingness is greater than this. In this case the number of imputations is equal to the percentage missingness. Multiple imputation is also performed on missing covariate and outcome data for all secondary clinical analyses in a similar way to the primary analysis.

Secondary outcomes are analysed using a suitable mixed-effects model (linear mixed-effects model for continuous outcomes, and a Poisson generalised linear mixed-effects model for count data) or accounting for the clustering using robust Huber-White standard errors. Secondary clinical endpoints include the change in Carer Strain Index, the number of seizures and the other subscales of the ELDQoL measurement (side effects, mood and behaviour). The covariates used for the analysis of the secondary outcomes are the same as those included in the primary outcome measure.

#### Economic analyses

A cost-utility analysis is used to evaluate effectiveness. The cost-utility analysis will allow comparison of cost-effectiveness against commonly accepted thresholds of acceptability in terms of the cost per quality-adjusted life-year (QALY) gained [38]. However, given the potential for a lack of sensitivity of the EQ-5D-5 L to benefits of the intervention for carers and for participants, we will also undertake a cost-benefit analysis. The time horizon of each evaluation is 6 months and the primary perspective is Health and Social Services.

A sensitivity analysis will consider a broader societal perspective. Cost differences across the two arms of the trial are estimated from questionnaires assessing provision of health and social care at baseline and after 6 months, and from time diaries completed by ENs. QALY gains are estimated at 0.5* health-related quality of life (QoL) at 6 months after controlling for QoL at baseline, ELDQoL-SSS, age, level of ID, mean number of seizures per month (calculated as above), and living circumstances (as above). Willingness-to-pay data are collected as open-ended responses with the use of prompt cards showing a range of values. Bootstrapping is used to estimate uncertainty in mean cost and outcomes, and to facilitate construction of cost-effectiveness acceptability curves for the cost-utility analysis. Bootstrapping refers to a statistical technique to estimate uncertainty around a parameter without assuming a parametric distribution for the population distribution of that parameter.

#### Qualitative analysis

The semi-structured interviews are analysed using a systematic process of indexing - thematic framework development; charting; mapping and interpretation. We will examine how the competency framework, compared to treatment as usual, impacts on relationships between the EN and family/paid carers with respect to (i) reported perceptions of patient health and quality of life, (ii) the involvement of patients in treatment decisions and (iii) the active engagement of carers with clinical epilepsy services.

### Safety procedure

It is not expected that there are any major negative effects on participants resulting from participating in this trial. No clinically indicated treatment is withheld in either arm of the trial. However, serious adverse events which might be expected include an increase in seizure frequency or severity, occurrence of uncontrolled seizures requiring paramedic support or hospital admission and emergence of anti-epileptic drug-related adverse effects.

The Principal Investigator (PI) at each cluster site is responsible for recording all serious adverse events and reporting them to the Chief Investigator (CI), via the Trial Coordinator, on trial serious adverse events forms. The Chief Investigator will then report all serious adverse events to the sponsor. Any serious adverse events classified as ‘related’ and ‘unexpected’ will be reported to the main Research Ethics Committee (REC) within 15 days of becoming aware.

## Ethics, dissemination and patient and public involvement

### Research ethics approval

This protocol has received ethical approval from the National Research Ethics Service (London Queen Square Committee) and, for Scotland, the Scotland A Research Ethics Committee. Amendments are reported to all study sites and the trial oversight committees. In order to enable inclusion of adults lacking capacity to decide whether to participate in research, appropriate approvals are sought from family or care providers, in line with ss. 30-34 of the Mental Capacity Act (England and Wales 2005) [39] or section 51 of the Adults with Incapacity Act (Scotland 2000) [40]. Those for whom consent/assent is not obtained will not participate in the study and will continue to receive their standard management.

### Consent/assent

All participants’ family carers or paid support workers and, where possible the participants themselves, are informed about the potential risks and benefits of the study. Study information is presented in terms of a written information sheet and also as an accessible ‘easy-read’ information sheet. Informed written consent is obtained for all participants. In England and Wales either the participant provides consent or if capacity to consent is absent, written assent is provided by a consultee. In Scotland informed consent is given either by the participant or, if they lack capacity, their nearest relative, welfare attorney or welfare guardian.

### Confidentiality

The Chief Investigator acts as custodian for the trial data. The following guidelines are strictly adhered to:

- Patient data are anonymised on the eCRF and paper CRF.
- All anonymised data are stored on a password-protected computer

### Patient and public involvement

All research studies funded by the UK NIHR are required to demonstrate good-quality patient and public involvement (PPI). The patient group involved in the EpAID study present difficulties in terms of representation as individuals with severe intellectual disabilities are more likely to have epilepsy. They are thus more likely to make up a large proportion of the participant group recruited for this trial. The Trial Advisory Group (TAG) for this study has attempted to overcome these challenges and ensure the views of those with severe ID are represented, along with their families and paid carers and the general public, through the experience and expertise of its members.

TAG members include the mother of a young woman with a severe ID and complex epilepsy and a home manager with experience of care for people with ID and epilepsy. The advisory group also consists of a representative from the charity Epilepsy Action who is able to consult through volunteer and adviser contacts and find family carers or members of the public to give views about specific questions as they arise. Joining the TAG are a Learning Disability Nurse with experience in the management of epilepsy, the trial CI, Co-ordinator, Research Assistants and other members of the research team as required.

The group address issues relevant to recruitment, retention and dissemination of findings. They advise on how to interact with participants and carers and help to make sure that important outcomes are not overlooked. Meetings happen at intervals of approximately 3 months but remain flexible to incorporate each stage of the research process. Advice gained through the TAG is acted upon and reported by the research team throughout the trial.

## Discussion

The EpAID Trial investigates whether an EN-led epilepsy management programme is effective and represents good value for money for people with an ID and epilepsy compared to current management (treatment as usual).

The trial methodology brings both strengths and weaknesses. Cluster randomisation protects against contamination [41]. Within each cluster the EN(s) will manage participants using just one approach. There are 16 individual cluster sites across England, Scotland and Wales, increasing the external validity of the trial. The inclusion of multiple different healthcare delivery organisations (NHS Trusts) within the trial increases the diversity of the population being studied, increasing the generalisability of the study.

However, the complex intervention to be trialled cannot be delivered and assessed in a double-blind manner [42]. Although participants will not be told which arm of the trial they are in, it is possible that they and their caregivers could notice a difference in their management. Moreover, the Hawthorne effect has the potential to influence this study, as clinicians in both arms may try to improve the management they provide for patients involved in the study [42], possibly overshadowing effects of the intervention.

Fidelity of delivery of the intervention is assessed by examination of activity diaries completed by the ENs. ENs could have large numbers of diaries to fill, so there is a chance that information could be omitted or forgotten. Previous studies have found this not to be the case [31, 32]. However, these studies did not have the volume of participants included in the EpAID Trial and it is possible that this could be detrimental to the accuracy and consistency of diary entries.

The following steps to minimise attrition are included as part of the trial design; (i) participation in the trial makes very few active demands on the participants, (ii) participants are eligible because they have ongoing seizures, meaning that they are likely to be agreeable to remaining in the trial, (iii) participation in the trial does not preclude receipt of any other clinically indicated treatment, (iv) the research team follows a flexible participant-led approach to gathering baseline and follow-up data – with contacts being at times and locations suggested by participants and respondents, and with, as far as possible, contacts being either face-to-face, by telephone or by post as preferred by respondents. There are, however, issues that may arise when using telephone contact to complete questionnaires. Questionnaire interviews conducted over the phone may be more readily misunderstood than face-to-face interviews, even if the participant has a copy of the relevant questionnaire in front of them (which may not always be possible). On the other hand, telephone calls may be more convenient for participants and carers as they can be scheduled around the needs of the participant, reducing the risks of failure or delay in obtaining baseline or follow-up measures.

There is a growing importance in evaluating the cost-effectiveness of clinical interventions alongside their efficacy [43]. This allows decision-makers to prioritise investment in a manner which reflects efficiency as well as equity concerns [44]. This importance is recognised by the EpAID Trial, which will involve two complementary economic evaluations: a cost-utility analysis capturing gains in participant QoL and allowing comparison against accepted thresholds (£20,000 per QALY), and a cost-benefit analysis that has the scope to capture a broader range of benefits to participants and their carers. These evaluations will provide the necessary data for commissioners of health services to assess whether to implement an EN service given the constraints of a limited budget.

The EpAID Trial investigates treatments for epilepsy in adults who also have an intellectual disability. As noted in the introduction, there is a clear need for research to improve epilepsy management in this group. However, undertaking a clinical trial in this population brings several challenges that need to be overcome. These include recruiting individuals who lack capacity to provide informed consent to participate and identifying appropriate consultees for these individuals. Some individuals have been in residential care for many years, occasionally with limited or no contact with family members. To overcome these challenges we work closely with the community teams who deliver care to this patient group, and through them, aim to constructively involve carers, particularly family carers. The importance of this approach is illustrated in a previous qualitative study of parents of adults with ID and epilepsy, which observed that, in their role as gatekeepers for access by health services to their grown-up children, the extent to which parents facilitated their offspring’s participation in therapeutic activities depended on the parents’ views, as opposed to clinicians’ views, of those activities [10].

Further issues may be related to participants being in group homes. Informed consent can be more difficult to gauge when the participant is reliant on others. The participant’s choices may be limited – they may be used to saying ‘yes’ or ‘no’ to every option [45], which clouds their ability to make a judgement related to informed consent. Furthermore, the researcher may be seen as an authority figure, leading to acquiescence – the person in authority makes a decision and the participant simply goes along with it [46].

The EpAID research could have important implications for ID and epilepsy services. If the intervention is found to be effective and cost-effective it will improve epilepsy outcomes for service users with ID, as they will receive more effective treatments. If on the other hand the intervention is found to be no more effective than current treatments, this knowledge will enable service commissioners and managers to deploy clinicians to other roles.

## Trial status

The study began in October 2013, recruitment commenced in October 2014.

### Trial protocol

Date 30 July 2015, Version 1.5.

## Abbreviations

CI, Chief Investigator; CTU, Clinical Trials Unit; DMEC, Data Monitoring and Ethics Committee; eCRF, Electronic case report form; ELDQoL-SSS, Epilepsy Learning Disability Quality of Life-Seizure Severity Scale; EN, Epilepsy Nurses; EpAID, Epilepsy And Intellectual Disability; GCP, Good clinical practice; ICC, Interclass correlation coefficient; ID, Intellectual disability; ISRCTN, International Standardised Randomised Controlled Trial Number; NHS, National Health Service; NIHR, National Institute of Health Research; PI, Principal Investigator; QALY, quality-adjusted life-year; QoL, Quality of life; REC, Research Ethics Committee; TAG, Trial Advisory Group

## Additional file

Additional file 1: EpAID Trial SPIRIT checklist. (XLSX 11 kb)
